# Toll-Like Receptors and Myocardial Inflammation

**DOI:** 10.4061/2011/170352

**Published:** 2011-09-29

**Authors:** Yan Feng, Wei Chao

**Affiliations:** Department of Anesthesia, Critical Care and Pain Medicine, Massachusetts General Hospital, Harvard Medical School, Boston, MA 02114, USA

## Abstract

Toll-like receptors (TLRs) are a member of the innate immune system. TLRs detect invading pathogens through the pathogen-associated molecular patterns (PAMPs) recognition and play an essential role in the host defense. TLRs can also sense a large number of endogenous molecules with the damage-associated molecular patterns (DAMPs) that are produced under various injurious conditions. Animal studies of the last decade have demonstrated that TLR signaling contributes to the pathogenesis of the critical cardiac conditions, where myocardial inflammation plays a prominent role, such as ischemic myocardial injury, myocarditis, and septic cardiomyopathy. This paper reviews the animal data on (1) TLRs, TLR ligands, and the signal transduction system and (2) the important role of TLR signaling in these critical cardiac conditions.

## 1. Introduction

Innate immune system represents the first line of defense against foreign pathogens. Toll-like receptors (TLRs) belong to the family of pattern recognition receptors (PRRs). PRRs recognize the conserved motifs in pathogens termed pathogen-associated molecular patterns (PAMPs) and trigger innate immune response [1, 2]. In addition to participating in the host defense against infectious pathogens, accumulating evidence suggests that TLRs also play an essential role in tissue inflammationand contribute to “noninfectious” tissue damage such as cardiac ischemia/reperfusion (I/R) injury, postischemic remodeling, and atherosclerosis [3–6]. Thus, understanding TLR signaling and their role in cardiovascular diseases may help to identify potential targets for intervention and have important clinical implications. This paper reviews TLR signaling and its critical roles in several inflammatory cardiac conditions: I/R injury, viral and autoimmune myocarditis, and septic cardiomyopathy.

## 2. Toll-Like Receptors

Toll means “amazing” and “fantastic” in German. In 1985, Anderson and colleagues coined it for a protein critical for early embryonic development of Drosophila [7, 8]. A decade later, Lemaitre et al. found that this protein was also essential to the host innate immunity against fungal infection in adult flies [9]. Subsequently, Medzhitov and colleagues identified a mammalian homologue of the Drosophila Toll protein in human and termed it “Toll-like receptor” [10]. Stimulation of TLR signaling leads to the activation of transcription factors such as NF-*κ*B, one of the most important proinflammatory transcription factors. To date, 11 human and 13 mouse TLRs have been cloned. TLR1-TLR9 are conserved in both human and mouse, and all of them are functional to recognize diverse ligands [2]. However, mouse TLR10 has no function due to a retrovirus insertion, whereas human TLR10 may function as a TLR2 coactivator [2, 11]. Finally, TLR11, TLR12, and TLR13 are present in mouse but lost in human [2].

**Figure 1:**
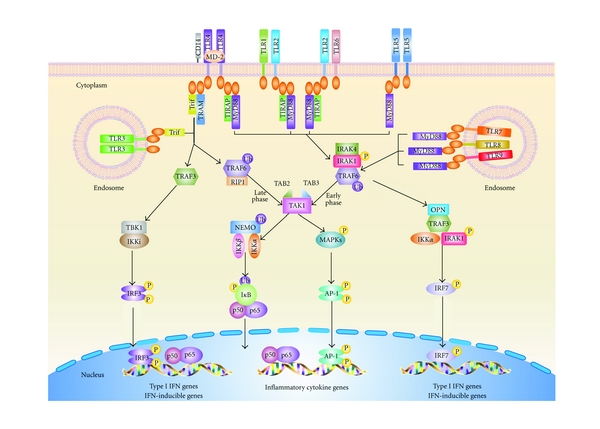
TLR signaling pathways. Upon respective ligands binding, TLRs form homo- or heterodimers and recruit one or more adaptor proteins, namely, MyD88, MAL/TIRAP, TRIF, or TRAM, to the cytoplasmic domains of the receptors through homophilic interactions between Toll/IL-1 receptor (TIR) domains present in each receptor and each adaptor. All TLRs with exception of TLR3 use the common MyD88-dependent pathway. TIRAP acts as a bridge to recruit MyD88 to TLR2 and TLR4 signaling, whereas TRIF is used in TLR3 signaling and, in association with TRAM, in TLR4 signaling. In MyD88-dependent pathway, MyD88 associates with IRAK4, IRAK1, and/or IRAK2. IRAK4 in turn phosphorylates IRAK1 and/or IRAK2 and promotes their association with TRAF6, which serves as a platform to recruit and activate the kinase TAK1. Activated TAK1 activates the IKK complex, composed of IKK*α*, IKK*β*, and NEMO (IKK*γ*), which in turn catalyzes phosphorylation and subsequent degradation of I*κ*B. I*κ*B degradation lets NF-*κ*B (*i.e.,* p50/p65) free to translocate from the cytoplasma to the nucleus, where it activates multiple gene expression. The transcription factor IRF7 is activated as the downstream signaling molecule of TLR 7, 8, and 9. It is directly phosphorylated by IRAK1 and then translocates into the nucleus to induce the expression of type I IFN and IFN-inducible genes. In the Trif-dependent pathway, Trif interacts with TRAF3 to activate TBK1 and IKKi, resulting in the dimerization and activation of IRF3, which then translocates into the nucleus activating the transcription of type I IFN and IFN-inducible genes.

TLRs are type I single-spanning membrane glycoproteins with a leucine-rich repeat of extracellular domain, which mediates ligand recognition, and an intracellular TIR domain, which recruits adaptors and activates downstream signaling. According to the ligands and the subcellular location, TLRs can be divided into two subgroups (Figure 1). TLR1, TLR2, TLR4, TLR5, TLR6, and TLR11 are located primarily on the cell surface and recognize mainly microbial membrane components such as lipids, lipoproteins, and proteins. On the other hand, TLR3, TLR7, TLR8, and TLR9 reside on the membranes of intracellular compartments, such as endosomes, lysosomes, endolysosomes, and endoplasmic reticulum, and are responsible for the recognition of microbial nucleic acids [2, 12]. 

### 2.1. TLR Ligands: PAMPs versus DAMPs

As summarized in Table 1, TLRs consist of a family of receptors that specifically bind to a wide range of pathogens including bacteria, fungi, parasites, and viruses through “PAMPs” recognition [1, 2]. Accumulating evidence has indicated that TLR can also act as a stress sensor in response to noninfectious tissue injury and recognize a variety of endogenous stress molecules through “DAMPs” recognition [13].

**PAMPs**

TLR4 was first identified as the receptor for LPS, a component of outer membrane of Gram-negative bacteria [34, 35]. Its extracellular domain forms a complex with MD-2 and serves as the main LPS-binding site [55]. Additional proteins including LPS-binding protein and CD14 are also involved in modulating LPS binding [56, 57]. TLR2 is the most diverse TLR that recognizes a large number of PAMPs, such as lipopeptides from diverse bacteria [14], peptidoglycan [16, 17] and lipoteichoic acid [17] from Gram-positive bacteria, LPS from certain Gram-negative bacteria [21], lipoarabinomannan from mycobacteria [23, 24], zymosan from fungi [26], glycosylphosphatidylinositol anchors from Trypanosoma cruzi [28], and hemagglutinin protein from measles virus [30]. It usually forms heterodimers with TLR1 or TLR6. In general, TLR1/2 recognizes triacylated lipopeptides [58], whereas TLR2/6 heterodimer recognizes diacylated lipopeptides [59]. TLR5 recognizes flagellin from bacterial flagella [45], and TLR11 recognizes profilin-like molecule from the protozoan parasite Toxoplasma gondii [53] and response to uropathogenic bacteria [54]. TLR3 senses dsRNA [31], synthetic analog of dsRNA, such as poly(I:C) [31], and certain small interfering RNAs [33]. It initiates antiviral immune responses through the expression of type I IFN and other inflammatory cytokines. TLR7 [46] and TLR8 [50] sense ssRNA from RNA viruses, imidazoquinoline compounds such as imiquimod and resiquimod (R-848) [47] and guanine analogs [49]. TLR9 senses unmethylated dinucleotides CpG DNA motifs, which are commonly present in bacteria and viruses but lacking in mammalian cells [51].

**DAMPs**

These endogenous ligands are ECM fragments or intracellular molecules produced either through release from preformed precursor or by *de novo* synthesis in response to tissue injury. DAMP-activated TLR signaling reportedly plays an important role in the pathogenesis of many inflammatory and autoimmune diseases. This topic is well reviewed by Piccinini and Midwood [13].

HSP60 was the first endogenous molecule linked to TLRs. Ohashi and colleagues found that similar to LPS, HSP60-induced TNF*α* expression and nitric oxide production were blocked in bone marrow-derived macrophages isolated from TLR4-deficient mice (C3H/HeJ strain) [36]. Since then, an increasing list of endogenous molecules has been identified to function as TLR ligands [2, 11, 13], including intracellular molecules released to extracellular environment after tissue injury, such as HSPs including HSP60 [15], HSP70 [18, 19, 37], HSP72 [38], HSP22 [39] and gp96 [20], and HMGB1 [22]. Others are ECM molecule such as fibronectin [40], biglycan [25], tenascin-C [41], versican [27], and fragments of ECM including oligosaccharides of hyaluronan [42], lower molecular weight hyaluronan [29, 43], and heparan sulfate [44]. In addition, chromatin-DNA and ribonucleoprotein complexes released from injured cells can activate intracellular TLRs. For example, mRNA exposure induces NF-*κ*B activation and IL-8 production in stable TLR3-expressed HEK 293 cells. Meanwhile, TLR3 specific-antibody suppresses the activation of dendritic cells after stimulation with *in vitro* transcribed RNA or endogenous RNA released from necrotic cells [32]. In systemic lupus erythematosus, plasmacytoid dendritic cells could be activated to secrete type I IFN by RNA sequences through TLR7 and TLR8 [48]. Moreover, the ability to activate rheumatoid factor B cells in response to IgG2a-chromatin immune complexes was abolished in MyD88^−/−^ mice, and the autoimmune complexes-induced activation was blocked by various inhibitors of TLR9 signaling [52].

### 2.2. TLR Signaling Pathways

As illustrated in Figure 1, upon activation, TLRs form dimers and initiate the downstream intracellular signaling. Heterodimerization occurs between TLR2 and TLR1 or TLR6 and between TLR4 and MD-2, whereas the other TLRs form homodimers. Ligand-induced homo-hetero dimerization of TLRs triggers the cytoplasmic signaling domains of the receptor to dimerize. The resulting TIR-TIR complexes trigger specific biological responses by initiating downstream signaling through a set of specific adaptors. So far, 5 adaptors have been identified [60]. They are MyD88, TIRAP, Trif, TRAM, and SARM [61]. TLRs interact with their respective adaptors *via* their TIR domain and the homologous domain present in these adaptors. Depending on the adaptors recruited, TLRs signaling can be divided into two distinct pathways: MyD88-dependent and Trif-dependent pathways. Mal acts as a bridge adaptor to help MyD88 recruiting to TLR2 and TLR4, whereas TRAM functions as a sorting protein that recruits Trif to TLR4 [2, 61].

**MyD88-Dependent Pathway**

MyD88-dependent pathway is activated by all TLRs with exception of TLR3. MyD88 signaling leads to inflammatory cytokine production by activating the transcription factor NF-*κ*B and MAPKs. MyD88 recruits IL-1 receptor-associated kinases (IRAKs), such as IRAK1, IRAK2, IRAK4, and IRAK-M. IRAK4 is activated initially and followed by the activation of IRAK1 and IRAK2, leading to an interaction with TRAF6 [2]. The IRAK1-TRAF6 complex then activates TAK1 through a process involving cytosol translocation of TAK1 and two regulatory components TAK-binding protein 2 (TAB2) and TAB3 and the ubiquitination of TRAF6. Activated TAK1 then phosphorylates IKK*β*, leading to phosphorylation and degradation of I-*κ*B, which releases NF-*κ*B and results in the nuclear translocation and DNA binding of NF-*κ*B [2].

**Trif-Dependent Pathway**

Trif-dependent pathway is utilized by TLR3 and TLR4. It induces type I IFN and inflammatory cytokines through the activation of the transcription factor interferon regulatory factor 3 (IRF3) and NF-*κ*B. Trif associates with TRAF3 and TRAF6. TRAF3 links a signaling complex involving the noncanonical IKKs, TRAF family member-associated NF-*κ*B activator (TANK) binding kinase-1 (TBK1) and IKKi, which catalyze phosphorylation of IRF3 and induce its nuclear translocation and type I IFN expression. Moreover, Trif also recruits TRAF6 and receptor-interacting protein 1 (RIP1), with the help of TAK1, leading to the activation of NF-*κ*B and MAPKs through ubiquitination-dependent mechanism similar to MyD88-dependent pathway [2, 12].

Of note, TLR4 reportedly activates both MyD88- and Trif-dependent pathways. After LPS binding, TLR4 initially triggers MyD88-dependent pathway on the plasma membrane and subsequently undergoes dynamin- and clathrin-dependent endocytosis and translocates to the endosome [2, 62]. This translocation is not only involved in degradation of TLR4, but also required for initiating Trif-dependent pathway [2, 62], which leads to IRF3 activation as well as late-phase activation of NF-*κ*B [2, 62, 63].

## 3. TLR and Ischemic Myocardial Injury

TLRs are highly conserved and expressed ubiquitously throughout species including mammals, chicken, flies, and plants. In mammals, they are expressed differentially in immune cells such as monocytes/macrophage [64], neutrophil [65, 66], natural killer cells [67], dentritic cells [68], mast cells [69], specific T and B lymphocytes [70, 71], and nonimmune cells, such as epithelial cells [72], skin keratinocytes [73], fibroblasts [74], and cardiomyocytes and endothelial cells in the heart [75–77]. Gene expression of TLR2, TLR3, TLR4, TLR5, TLR7, and TLR9 has been reported in mouse heart tissue and in cardiomyocyte cell line [75–77]. Signaling* via* TLR2, TLR4, and TLR5, but not TLR3, TLR7, or TLR9, can initiate proinflammatory cytokines expression and inhibit cardiomyocyte contractility [75, 78]. Moreover, the mRNA expression of all 10 TLRs has been identified in the human heart tissue [79]. The one with highest expression is TLR4, whereas the lowest ones are TLR8, TLR9, and TLR10. 

While tissue hypoxia is the initial cause of myocardial injury during transient ischemia, reperfusion-induced myocardial inflammation is an important contributor to ischemia-induced myocardial injury [80]. In fact, innate immune response is by far the most common cause of myocardial inflammation after I/R, characterized as proinflammatory cytokine release, endothelial cell activation, complement deposition, inflammatory cell infiltration, and increased vascular permeability [81–83]. Many of these inflammatory responses are regulated by NF-*κ*B signaling pathway [84, 85]. Since TLRs are important upstream activators of NF-*κ*B signaling, the role of TLRs in cardiac ischemic injury has been intensely studied in the past 10 years [3]. Among those TLRs expressed in the heart, TLR2 and TLR4 have been most investigated (Table 2). 

### 3.1. TLR2

Several studies have indicated that TLR2 signaling is involved in myocardial I/R injury [86–89]. In an *ex vivo* model of I/R, TLR2^−/−^ mice exhibited improved LV function compared to WT mice following I/R [86]. TLR2 is also involved in coronary artery endothelial dysfunction with impaired vessel relaxation induced by transient ischemia [87]. Similar to TLR4-deficient animals, TLR2^−/−^ mice had reduced inflammatory responses and smaller MI sizes after I/R compared to WT control. Moreover, using chimeric TLR2 deletion models, Arslan and coworkers demonstrated that leukocyte TLR2 played a prominent role in mediating myocardial injury during I/R. They found that WT mice with circulatory cells derived from TLR2^−/−^ mice were protected from I/R injury [88]. Administration of an anti-TLR2 antibody prior to reperfusion reduced MI sizes, preserved cardiac function, and decreased scar formation. Importantly, these cardiac benefits in TLR2^−/−^ mice were associated with persistent attenuation of myocardial inflammation, such as reduced leukocytes infiltration and attenuated proinflammatory cytokines production. Interestingly, chemokines and adhesion molecules, which are essential for recruiting leukocytes to ischemic myocardium, were not changed.

### 3.2. TLR4

Several studies have demonstrated that TLR4 plays an important role in mediating immune cells infiltration, cytokine production, and complement activation during I/R. Oyama and colleagues [90] first demonstrated that after transient ischemia (1 h of coronary artery occlusion and 24 h of reperfusion), TLR4-deficient mice, C57/B10 ScCr and C3H/HeJ, had significantly smaller MI sizes with more than 50% reduction compared to their respective control mice, C57/BL10 ScSn and C3H/OuJ. C57/B10 ScCr mice have natural TLR4 gene deficiency, whereas C3H/HeJ mice have a spontaneous missense point mutation in the TIR domain. Furthermore, the decreased myocardial infarction in TLR4-deficient mice was associated with attenuated myocardial inflammation as evidenced by fewer neutrophil infiltration, less lipid peroxides production, and less complement 3 deposition in the heart [90]. 

In a similar, but shorter, *in vivo* protocol (1 h of ischemia followed by 2 h of reperfusion), Chong and colleagues [91] independently demonstrated a cardiac protection in C3H/HeJ mice with 40% reduction of MI compared to WT mice. I/R induced significant activation of ERK, p38 MAPK, and JNK, and translocation of NF-*κ*B and AP-1 in WT mice. However, in C3H/HeJ mice, there was a significant reduction in JNK and NF-*κ*B/AP-1 activity and mRNA levels of myocardial IL-1*β*, IL-6, and MCP-1 [91]. Moreover, blunting TLR4 signaling by eritoran, a specific TLR4 antagonist, also resulted in decreased MI sizes and attenuated myocardial inflammatory responses, such as reduced JNK phosphorylation, attenuated NF-*κ*B nuclear translocation, and decreased gene transcripts of TNF*α*, IL-1*β*, IL-6, MCP-1, MIP-1*α*, and MIP-2 [92].

TLR4 may also mediate systemic cytokine production following myocardial I/R injury. Kim and colleagues measured the protein level of proinflammatory cytokines in the myocardium and serum after I/R [93]. They noticed a robust increase in the serum levels of TNF*α*, IL-1*β*, and IL-6 in response to 1 h of ischemia and 2 h of reperfusion *in vivo*. TLR4 deletion led to significant reduction in systemic inflammation, but only selective reduction in myocardial IL-6, and reduced MI sizes [93]. The authors thus speculated that systemic rather than local inflammatory response involving TLR4 signaling contributes to I/R injury. 

In an isolated heart model of global I/R, which is devoid of circulating cells or other blood components, Cha and colleagues found that TLR4-deficient hearts had reduced levels of TNF*α* and IL-1*β* and improved cardiac contractile function compared to WT hearts [94]. Administration of TNF*α* and IL-1*β* to TLR4 defective heart, however, abrogated the beneficial effect of functional recovery in TLR4-deficient hearts after global ischemia [94], whereas functional recovery after ischemia was also improved in TNF*α*- and IL-1*β*-deficient hearts, as well as in wild-type hearts treated with TNF-binding protein or IL-1 receptor antagonist. These studies suggest that myocardial TLR4 signaling may contribute to cardiac dysfunction *via *TNF*α*- and IL-1*β*-dependent mechanisms after global I/R [94]. Interestingly, in a similar *ex vivo* model of I/R injury, Meng and colleagues found that 70-kDa heat shock cognate protein was released from ischemic myocardium and mediates, *via* a TLR4-dependent mechanism, myocardial NF-*κ*B activation and cytokine/chemokine production in response to I/R [95, 96]. 

TLR4 signaling may also mediate inflammatory response and contribute to myocardial injury during heart transplantation. In a mouse model of heart transplantation, Kaczorowski and colleagues [97, 101] demonstrated that the serum myocardial injury marker, troponin I, was markedly increased in the recipient mice. This was associated with elevated serum inflammatory cytokines, such as TNF*α*, IL-1 *β*, IL-6, and MCP-1. Similarly, myocardial inflammation was also dramatically induced in the graft. However, all of these inflammatory responses were attenuated in TLR4-deficient mice subjected to the same transplantation protocol, suggesting that TLR4 signaling mediates myocardial injury and systemic and local inflammation during the transplantation.

### 3.3. MyD88

**Figure 2:**
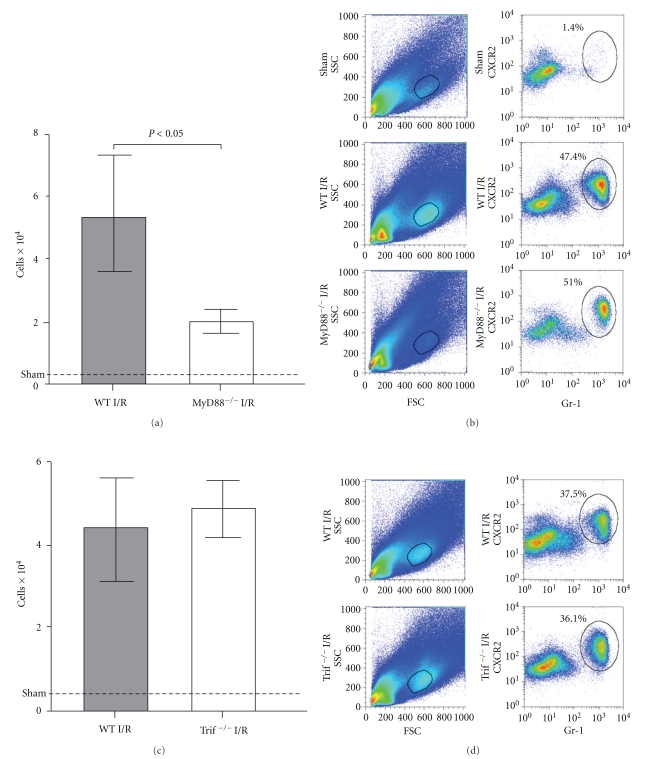
Myocardial neutrophil recruitment after I/R in MyD88^−/−^ and Trif^−/−^ mice. Twenty-four hours after 60 min of left anterior descending coronary artery (LAD) ligation, the hearts were isolated, perfused, and digested. After removal of the large cardiomyocytes through filtration, 50% of total cells were loaded onto flow cytometry and gated on Gr-1 and CXCR2. (a) Total Gr-1+ cells as measured by flow cytometry from the hearts subjected to I/R in MyD88^−/−^ mice. Each error bar represents mean ± SD of 4 mice. A small number of neutrophils were recovered from the sham-operated hearts as indicated by the line. (b) A representative example of flow cytometry plots of myocardial infiltrating cells from sham, WT-I/R, and MyD88^−/−^-I/R mice. (c) Total Gr-1+ cells as measured by flow cytometry from the hearts subjected to I/R in Trif^−/−^ mice. Each error bar represents mean ± SD of 3 mice. A small number of neutrophils were recovered from the sham-operated hearts as indicated by the line. (d) A representative example of flow cytometry plots of myocardial infiltrating cells from WT-I/R and Trif^−/−^-I/R mice. FSC, forward scatter; SSC, side scatter. (Feng et al., [100], used with permission).

**Figure 3:**
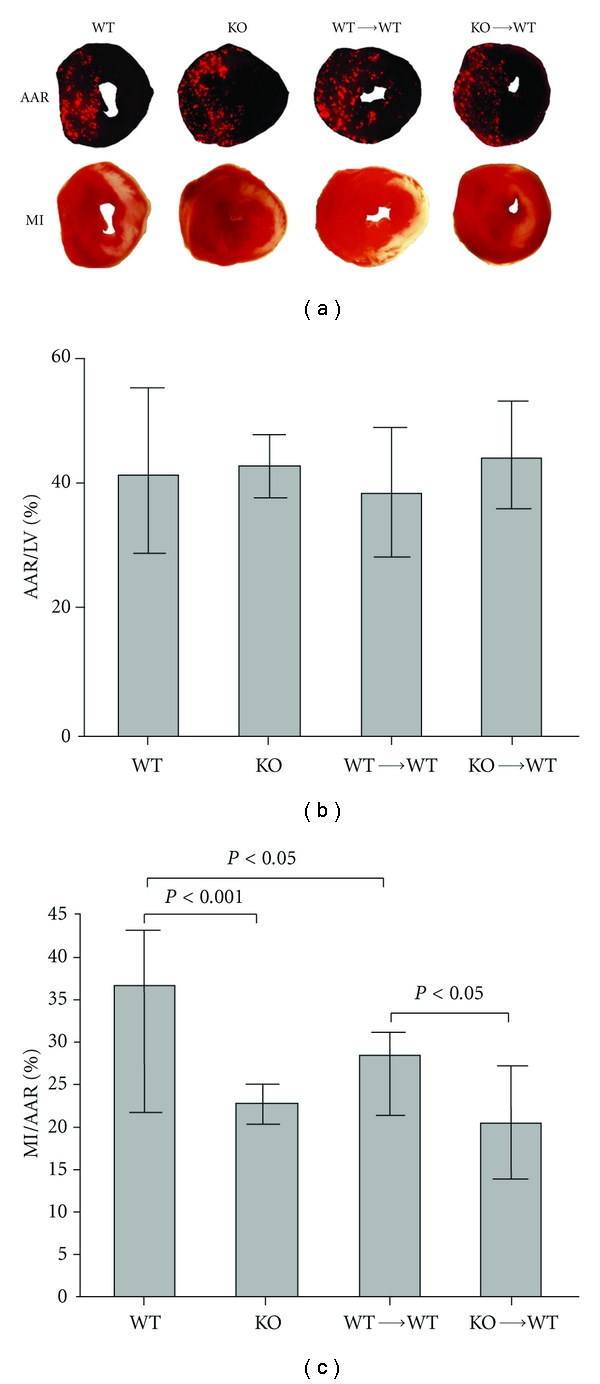
Decreased MI sizes in MyD88-knockout (KO) and KO→WT chimeric mice compared with wild-type (WT) and WT→WT chimeric mice. Mice were subjected to 30 min of ischemia and 24 h of reperfusion. At the end of reperfusion, animals were euthanized, and area-at-risk (AAR) and MI were analyzed. (a) Representative of triphenyltetra zolium chloride (TTC) staining (*bottom*) and fluorescent microsphere distribution (*top*) of myocardial sections from the 4 groups of mice. The nonischemic area is indicated by red fluorescent staining, area at risk (AAR) by area devoid of red fluorescent light, and infarct area by white. (b) Cumulative data of AAR/left ventricle (LV). (c) Cumulative data of MI/AAR. Each error bar represents mean ± SD of 6–9 mice. (Feng et al., [100], used with permission).

**Figure 4:**
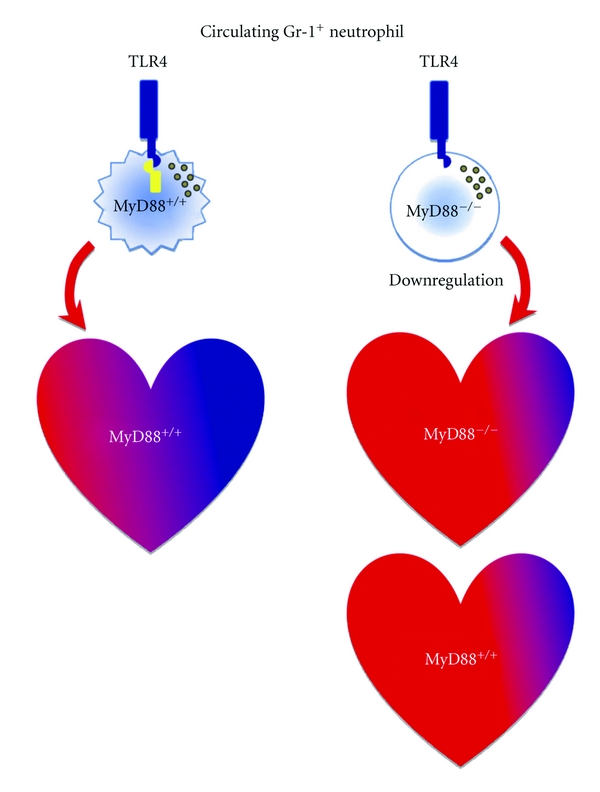
Schematic diagram of cardiac infarct size (blue region) after acute ischemia and reperfusion (I/R) and neutrophil CXCR2 downregulation by deletion of myeloid differentiation factor 88 (MyD88) globally (*right top*) and targeted to leukocytes only (*right bottom*). (Schmid-Schönbein, [103], used with permission).

Given its critical role in TLR signaling, it is not surprising that MyD88 also plays a role in mediating myocardial innate immune response and contributes to injury after I/R. Employing genetically modified mouse models or local transgene expression of dominant negative MyD88 (dn-MyD88), investigators demonstrate that MyD88 signaling participates in I/R-induced myocardial inflammation and myocardial infarction [98, 99]. In a rat model of I/R injury, Hua and colleagues reported that adenoviral expression of dn-MyD88 three days prior to the onset of myocardial ischemia led to reduced infarct sizes and attenuated NF-*κ*B activity, consistent with the notion that MyD88 signaling may contribute to ischemic myocardial injury by attenuating inflammatory response that is dependent on NF-*κ*B signaling. One issue with adenovirus-mediated gene expression in the myocardium, however, is the well-documented innate immune response that may cause local inflammation rather than attenuate it [102]. The challenge would be to separate I/R-induced inflammation from adenovirus-mediated innate immune response. In a mouse model of I/R injury, Feng and colleagues found that compared to WT mice, mice deficient in MyD88 had markedly reduced myocardial infarction and significantly improved LV function between day 1 and day 7 after transient ischemia as measured by transthoracic echocardiography [99]. MyD88^−/−^ mice also exhibited significantly reduced myocardial cytokines and chemokines [99, 100]. Flow cytometry analysis of cardiac cells isolated from the digested hearts demonstrated a robust increase in Gr-1+ neutrophils in the myocardium following I/R and a very small number of neutrophils in the myocardium of sham-operated mice. In contrast, there was a marked reduction in myocardial Gr-1+ neutrophils in MyD88^−/−^ mice (Figure 2). Using an *in vivo* migration assay, the investigators found that MyD88^−/−^ mice had markedly attenuated neutrophil migratory function, which was associated with decreased neutrophil CXCR2 expression and lower tissue KC, a neutrophil chemoattractant [100]. Interestingly, deletion of Trif, another innate immune adaptor, had no impact on myocardial neutrophil recruitment following I/R (Figure 2) or on neutrophil CXCR2 modulation [100]. In an effort to determine the specific contribution of myocardial MyD88 to cardiac injury following ischemia, Feng and colleagues tested whether or not MyD88 deficiency would have any effect on myocardial injury in isolated mouse hearts. Surprisingly, MyD88-deficiency had no significant impact on MI sizes and cardiac function in isolated hearts subjected to global I/R [99]. This finding is consistent with the notion that the cardiac benefits observed in MyD88^−/−^ mice *in vivo* may require circulating blood components during I/R. Further studies in chimeric MyD88 deletion models demonstrated that compared to WT mice or WT mice transplanted with MyD88^+/+^ bone marrow (WT*→*WT), WT mice transplanted with MyD88^−/−^ donor bone marrow (KO*→*WT) had significantly decreased MI sizes (Figure 3). Collectively, these findings suggest that MyD88 signaling is essential for maintaining neutrophil migratory function and chemokine receptor expression. MyD88 signaling in bone marrow-derived neutrophils may play a specific and critical role in the development of myocardial I/R-induced injury (Figure 4) [103].

## 4. TLR and Myocarditis

Myocarditis is defined clinically as inflammation of the heart muscle and has been identified as a major cause of sudden, unexpected death in adults less than 40 years of age and young athletes, accounting for approximately 20% of such cases. It is estimated that the incidence of myocarditis in the general population ranges from 1.06% to 5.0% [104–106]. The causes of acute myocarditis include infection with various pathogens (viral, bacterial, and fungi), autoimmune disorders, systemic diseases, drugs, and toxins.

**Viral Myocarditis**

Viruses are the predominant cause of myocarditis in North America and Europe, whereas Trypanosoma cruzi and Chagas' disease are the major contributors to the high incidence of myocarditis in South America. While the exact role of various TLRs in the pathogenesis of viral myocarditis and cardiomyopathy is yet to be defined, both protective and detrimental effects have been reported (Table 3).

### 4.1. TLR3

TLR3 recognizes dsRNA and is involved in viral recognition. Hardarson and colleagues found that compared to WT mice, TLR3-deficient mice were susceptible to encephalomyocarditis virus (EMCV) infection with higher mortality, increased myocardial viral load, and more severe myocardial injury [107]. Importantly, myocardial inflammatory cell infiltration and cytokine mRNA expression, such as TNF*α*, IL-1*β*, and IFN-*β*, were significantly attenuated and delayed in TLR3^−/−^ mice. These data suggest that EMCV infection induces a TLR3-dependent innate immune response in the heart, which represents a critical host protective mechanism against the virus-induced myocardial injury and mortality.

**Figure 5:**
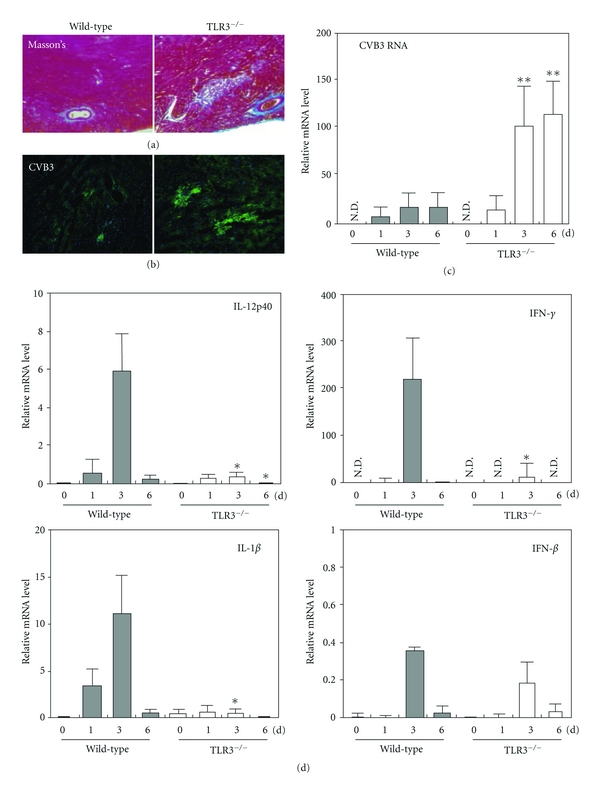
Myocarditis in TLR3^−/−^ mice infected with CVB3. (a) Histopathologic results of hearts collected from TLR3^−/−^ and wild-type mice 12 days after CVB3 infection was evaluated with Masson's trichrome staining. Data represent eight mice per group. (b) Immunofluorescence of hearts from TLR3^−/−^ or wild-type mice 9 days after CVB3 infection followed by anti-CVB3 antibody staining. Data represent three mice per group. (c),(d) Real-time RT-PCR analysis of the expression of positive-strand CVB3 RNA (c) or the indicated cytokine genes (d) in hearts of TLR3^−/−^ and wild-type mice on the indicated days after CVB3 infection. Data are presented as mean ± SD of triplicate determinations. All experiments were performed more than twice with similar results. **P < 0.01; *P < 0.05. (Negishi et al., [108], used with permission).

A similar protective role of TLR3 was reported in CV-induced myocarditis [108]. In that study, Negishi and colleagues demonstrated that compared to WT mice, TLR3^−/−^ mice had higher mortality, higher systemic and myocardial viral replication, and depressed systemic as well as myocardial cytokine gene induction (IL-12p40 and IL-1*β*) after CV infection. Local myocardial production of IFN-*γ*, not IFN-*β*, was significantly reduced in TLR3^−/−^ hearts (Figure 5). These studies demonstrate that type II IFN rather than type I IFN plays a critical role in the antiviral responses of TLR3 signaling [108].

### 4.2. TLR4

TLR4 mRNA was reportedly increased in endomyocardial biopsy samples from patients with clinically suspected myocarditis and from those with idiopathic dilated cardiomyopathy. Immunohistochemical analysis revealed that TLR4 was mainly expressed in infiltrated leukocytes and cardiomyocytes. The increase in myocardial TLR4 mRNA expression was associated with enteroviral replication and cardiac dysfunction in human myocarditis [114]. In an animal model of myocarditis, investigators found that TLR4 and IL-12 receptor *β*1 exacerbated coxsackievirus replication and myocarditis, whereas IFN-*γ* protected against viral replication [111]. TLR4 signaling was also associated with increased proinflammatory cytokines (IL-1*β* and IL-18) expression in the infected hearts, suggesting these two cytokines play an important role in the pathogenesis of CV-induced myocarditis [111].

### 4.3. MyD88 and Trif

As noted above, MyD88 and Trif are two adaptors critical for TLR signaling, but their roles in the pathogenesis of viral myocarditis appear very much different. Fuse and coworkers found that within days after CVB3 inoculation, myocardial MyD88 and IRAK-4 expression was elevated. Moreover, compared to WT mice, mice deficient in MyD88 had less myocardial inflammation and injury, reduced CVB3 viral titers, and improved survival [113]. The myocardial cytokines (IL-1*β*, TNF*α*, IFN-*γ*, IL-10, and IL-18) was significantly decreased, but IFN-*α* and IFN-*β* were increased in MyD88^−/−^ mice. This study established MyD88 signaling as a major contributor to CVB-induced myocardial inflammation and as a critical regulator in myocardial viral replication possibly *via* type I IFN-dependent mechanism [113]. On the other hand, Trif is the key adaptor essential for TLR3 signaling. Similar to TLR3^−/−^ mice subjected to viral myocarditis, Trif^−/−^ mice reportedly also had higher viral load, attenuated cytokine gene expression than WT mice [108, 112], and marked increase in mortality after CVB3 infection [112]. The antiviral protection of Trif signaling was probably mediated by type I IFN-*β*, since myocardial IFN-*β* expression was markedly suppressed in Trif^−/−^mice and administration of IFN-*β* effectively reduced myocardial viral load and local inflammation and markedly improved the long-term survival rate in Trif-deficient animals [112].

**Autoimmune Myocarditis**

There is compelling evidence in a significant subset of patients with myocarditis and in several animal models of experimental autoimmune myocarditis (EAM) that host autoimmunity plays an important role in the pathogenesis of myocarditis and subsequent dilated cardiomyopathy [115]. TLR signaling activates the adaptive immune system by inducing proinflammatory cytokine production and upregulating costimulatory molecules of antigen presenting cells and is involved in autoimmune myocarditis.

In a mouse model of EAM, Nishikubo and colleagues [116] demonstrated that TLR4-induced Th1 immune response was required for the development of myocarditis induced by myosin and BCG. Similarly, in comparison to WT littermates, MyD88^−/−^ mice were protected from myocarditis after immunization with *α*-myosin heavy chain-derived peptide (MyHC-*α*) and complete Freund's adjuvant [117]. This protection against EAM is due to impaired expansion of heart-specific CD4+ T cells after immunization. The serine/threonine kinase PKC-*θ* is required for certain T cell-driven autoimmune responses such as myocarditis. Mice deficient in PKC-*θ* did not develop EAM. However, TLR9 activation by CpG could overcome the PKC-*θ* deficiency and restored EAM in PKC-*θ*-deficient mice by activation of T cells [118]. To determine the role of the intracellular TLRs in EAM, Pagni and colleagues induced experimental EAM in mice deficient in TLR3, TLR7, and TLR9 by immunization with MyHC-*α* and complete Freund's adjuvant. They found that myocardial cellular infiltration and *in vitro *proliferation of MyHC-*α*-restimulated splenocytes were markedly reduced in TLR7^−/−^ and MyD88^−/−^ mice, while TLR3^−/−^ and TLR9^−/−^ mice showed similar myocardial inflammatory cell infiltration as WT mice. These data suggest that TLR7 and MyD88 signaling mediates myocardial inflammation and injury during the EAM [109]. Zhang and colleagues reported that human cardiac myosin could act as an endogenous ligand to directly activate human monocytes to release proinflammatory cytokines. This effect of human myosin is TLR2 and TLR8 dependent [119].

## 5. TLR and Septic Cardiomyopathy

Sepsis is defined as the systemic inflammatory response syndrome that occurs during infection. It has an estimated prevalence of 751,000 cases each year in the United States, and over 210,000 of them die [120]. Sepsis is the 10th leading cause of death in the US [121]. Cardiovascular collapse induced by cardiac dysfunction and profound vasodilatation represents a main feature of septic shock and contributes to its high mortality. Since TLRs play an essential role in recognizing various microbial components such as LPS, lipoprotein, viral/bacterial DNA, these receptors play a pivotal role in the host innate immune defense and facilitate the adaptive immunity against foreign pathogens. On the other hand, inappropriate and imbalanced host immune response via TLR-dependent mechanisms may also contribute to the pathogenesis of sepsis. 

### 5.1. TLR2

**Figure 6:**
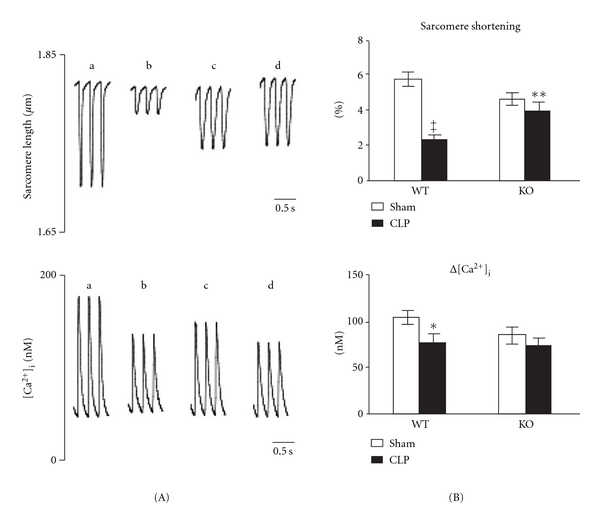
TLR2^−/−^ mice have improved cardiomyocyte function after polymicrobial sepsis. Wild-type (WT) and TLR2^−/−^ mice underwent sham or cecum ligation and puncture (CLP) procedures. Twenty-four hours later, the hearts were harvested and cardiomyocytes were isolated. A, Representative tracing of sarcomere shortening and Ca^2+^ transients in cardiomyocytes isolated from WT (a, b) and TLR2^−/−^ (c, d) mice subjected to either sham (a, c) or CLP (b, d) surgeries. B, Accumulated data of sarcomere shortening and Ca^2+^ transients. Each error bar represents mean ± SE. The data in each group were recorded from 16 to 27 single adult cardiomyocytes isolated from more than four mice. *P < 0.05 versus WT sham; **P < 0.01 versus WT CLP; ^‡^ *P* < 0.001 versus WT sham. KO, knockout (TLR2^−/−^). (Zou et al., [124], used with permission).

Knuefermann and colleagues [122] demonstrated that infusion of the Gram-positive bacteria *S*. *aureus* to isolated perfused heart activated myocardial IRAK-1 and NF-*κ*B signaling, increased TNF*α* and IL-1*β* production, and induced marked contractile dysfunction under an *ex vivo* condition. The cardiac effects of *S. aureus* was dependent on myocardial TLR2, since TLR2-deficient hearts were protected from the above inflammatory responses and myocardial dysfunction. Zhu and colleagues [78] demonstrated that peptidoglycan-associated lipoprotein, a naturally occurring TLR2 agonist and a ubiquitous Gram-negative bacterial outer-membrane protein that is shed by Gram-negative bacteria (e.g., *E. coli*) into the circulation of septic animals [123], induced pro-inflammatory cytokine production and directly inhibited cardiomyocyte function (sarcomere shortening and Ca^2+^ transients) *in vitro.* Zou and colleagues [124, 125] demonstrate that TLR2 plays a critical role in myocardial inflammation, ROS production, and cardiac dysfunction during bacterial sepsis. In a mouse model of polymicrobial sepsis (cecum ligation and puncture, (CLP)), these investigators found that compared to WT mice, TLR2^−/−^ mice had better survival, markedly improved cardiac function as measured by serial echocardiography, left ventricular pressure in isolated heart, and sarcomere shortening/Ca^2+^ transients in isolated cardiomyocytes (Figure 6), and depressed systemic and myocardial inflammatory cytokines production [124]. They further demonstrated that TLR2 activation by Pam3cys was sufficient to induce intracellular ROS production in neutrophils and cultured cardiomyocytes *in vitro* and that TLR2 deficiency markedly reduced intracellular ROS production in neutrophils isolated from polymicrobial peritoneal space [125]. While it remains unclear whether or not polymicrobial sepsis exerts cardiac dysfunction directly through TLR2 signaling *in vivo *[126], recent evidence appears to suggest that it is nonhematopoietic (parenchymal) TLR2 that plays a predominant role in mediating myocardial inflammation and cardiac dysfunction during polymicrobial sepsis [125] and as noted above, pathogenic ligand activation of TLR2 can induce direct functional depression of isolated cardiomyocyte *in vitro* [78].

### 5.2. TLR4

The role of TLR4 in sepsis-induced cardiac dysfunction has been studied mainly in endotoxemic models. LPS administration induces NF-*κ*B activation [127] that leads to robust myocardial cytokines expression, such as TNF*α*, IL-1*β*, and myocardial dysfunction [128, 129]. LPS also reportedly upregulates TLR4 and CD14. Mice deficient in TLR4, CD14, and IRAK-1 were protected from endotoxic shock with reduced myocardial inflammation and improved cardiac function [129–131]. It is unclear, however, whether or not LPS elicits its cardiac depressive effect directly through myocardial TLR4. A few studies suggest that LPS-induced cardiac dysfunction may be an indirect effect secondary to immune cell TLR4 activation. For example, Tavener and colleagues [132] found that cardiomyocytes isolated from LPS-treated mice exhibited reduced sarcomere shortening and Ca^2+^ transients, whereas *in vitro* treatment with LPS failed to inhibit cardiomyocyte function. Further studies in chimeric mice suggest that TLR4 in bone marrow-derived hematopoietic cells is probably responsible for cardiac dysfunction during endotoxic shock [132–134]. However, using similar chimeric models, Fallach and colleagues recently found that, mice deficient in TLR4 in bone marrow-derived cells, but not in parenchymal tissues, remain to be sensitive to LPS challenge. They suggest that cardiomyocyte, not hematopoietic, TLR4 contributes to cardiac depression during endotoxemia [135]. 

It should be pointed out that while endotoxin models are highly reproducible and can provide great insight into inflammatory processes [136], these ligand-based models lack an infectious focus and do not closely mimic the pathophysiology observed in septic patients. On the other hand, bacterial infection models such as CLP closely resemble the clinical scenario of sepsis such as bowel perforation. Importantly, the contribution of TLR4 signaling in the two models of sepsis may differ significantly. For example, studies have demonstrated that TLR4 deletion confers a survival protection against endotoxin shock [35, 137] but no survival benefit in CLP model [138]. These data suggest that host mobilizes different innate immune defense mechanisms in endotoxemia and polymicrobial septic peritonitis [138]. Moreover, recent data indicate that endotoxemia and CLP utilize different signaling pathways to induce cardiac dysfunction and systemic inflammation. For example, MyD88, but not Trif, plays a predominant role in mediating cardiac dysfunction, systemic inflammation, and mortality during CLP, whereas MyD88 and Trif are both important for systemic inflammation, cardiac depression and mortality during endotoxin shock [139]. These data clearly illustrate the critical difference in the role of TLR4 signaling in these two models of sepsis.

### 5.3. TLR5

Rolli and coworkers first demonstrated that bacterial flagellin, a TLR5 ligand, induced marked myocardial inflammation and contractile dysfunction [140]. In cultured H9c2 cells and in primary rat ventricular cardiomyocytes, flagellin was found to activate NF-*κ*B and MAPK and induce TNF*α* and MIP-2 expression. The flagellin-induced NF-*κ*B activation was TLR5-dependent. *In vivo* administration of flagellin led to myocardial NF-*κ*B activation, and expression of TNF*α*, IL-1*β*, IL-6, MIP-2, and MCP-1 increased myocardial neutrophil infiltration, and reversible cardiac dysfunction [140]. However, it is yet to be determined if TLR5 signaling plays a role in the pathogenesis of myocardial inflammation and cardiac dysfunction in more clinically relevant models of sepsis.

### 5.4. TLR9

Paladugu and colleagues [141] demonstrated that bacterial DNA and RNA derived from clinically pathogenic *S. aureus *and *E. coli *isolates induced a concentration-dependent depression of maximum extent and peak velocity of contraction of rat ventricular cardiomyocytes. Significant, but more modest, depression was also induced by a nonpathogenic *Escherichia coli *isolate. Pretreatment with DNase or RNase abrogated this effect. Similarly, *in vivo* administration of synthetic DNA (CpG-ODN) caused myocardial NF-*κ*B activation and inflammatory cytokine production (TNF*α*, IL-1*β*, and IL-6). *In vitro*, CpG-ODN inhibited sarcomere shortening of isolated mouse cardiomyocytes. Both the *in vivo* and *in vitro* effects of CpG were abolished in TLR9-deficient mice [142]. 

### 5.5. MyD88 and Trif

Using the CLP model [143] or a similar model [144], studies have established the critical role of MyD88 signaling in the pathogenesis of polymicrobial sepsis. In a colon ascendens stent peritonitis model, a highly inflammatory model, MyD88^−/−^ mice were found to be protected with improved survival and attenuated systemic inflammation within the first 48 hours [144]. However, in a CLP model with a low grade of severity of peritoneal polymicrobial sepsis, MyD88^−/−^ mice had worse survival compared with WT mice despite significantly attenuated systemic inflammation and reduced lymphocyte apoptosis in these mice [143]. In comparison, the role of Trif signaling in polymicrobial sepsis is not well understood. In a less severe sepsis model, Trif-deficient mice have reduced cytokine production including TNF*α*, IL-6, and IL-10 suggesting Trif signaling may contribute to systemic inflammation in a mild form of animal sepsis [143]. Feng and colleagues [139] compared the different role of MyD88- and Trif-signaling in endotoxemic and CLP models of sepsis. They demonstrate that MyD88 signaling is the dominant determinant in mediating inflammation, cardiac dysfunction, and mortality, whereas Trif signaling plays no major role, in the development of cardiac dysfunction and mortality in severe polymicrobial sepsis. But, as noted above, in endotoxemic model, MyD88 and Trif play an equally important role in mediating inflammation (IL-1*β*, IL-6, and TNF*α*), cardiac depression, and high mortality [139].

## 6. Summary and Perspective

During the past decade, studies characterizing the role of TLRs in the innate immunity and the immunopathology of human diseases have been extensive. A wide variety of microbial and nonmicrobial TLR ligands have been identified. These ligands act through their respective TLRs and elicit a variety of biochemical and proinflammatory responses *via* the distinct intracellular signal transduction systems. By medicating the critical and complex tissue inflammatory signaling, either protective or damaging in nature, TLRs play a pivotal role in the pathogenesis of cardiac critical conditions, such as acute ischemic myocardial injury, viral and autoimmune myocarditis, and septic cardiomyopathy. Several important future directions can be enumerated for characterization of the cellular and molecular mechanisms by which TLRs contribute to these cardiac conditions. While numerous studies have indicated the possible contributory role of TLRs in the development of ischemic myocardial injury, there are many unanswered questions that are critical for our ultimate understanding of the role of TLR signaling. For example, what are the specific contributions of *cardiac* versus *immune *cell TLRs to myocardial inflammation and infarction following I/R? How does the dual role of TLR4 signaling, *that is*, *proinflammatory* versus *antiapoptotic effect,* determine the final phenotypic outcome of myocardial injury [145]? How can we promote the protective *preconditioning *effect and at the same time prohibits the injurious *proinflammatory* effect of TLR signaling during myocardial ischemia [3]? Delineating these cellular and molecular details will help the future design of therapeutic strategy. Future studies will also be needed to delineate the role of *cardiac* versus *systemic* TLRs in the development of septic cardiac dysfunction and to define the intracellular mechanisms that control TLR-mediated deleterious cardiac dysfunction during sepsis. Without doubt, as we are developing new knowledge on the fine structure of the cellular and molecular mechanisms involved in these cardiac diseases, we will have better understanding on the essential role of TLRs in the human diseases. Dissecting the complex cellular and molecular pathways by which TLR signaling controls myocardial inflammation and cardiomyocyte injury will shed light on the mechanisms of these diseases and have significant clinical implications.

## Figures and Tables

**Table 1:** TLR ligands: PAMPs versus DAMPs.

TLRs	PAMPs	Pathogens	Ref.	DAMPs	Ref.
TLR2	Lipopeptides	Bacteria	[14]	HSP60	[15]
	Peptidoglycan	Gram+ bacteria	[16, 17]	HSP70	[18, 19]
	Lipoteichoic acid	Gram+ bacteria	[17]	Gp96	[20]
	LPS	Leptospira interrogans	[21]	HMGB1	[22]
	Lipoarabinomannan	Mycobacteria	[23, 24]	bioglycan	[25]
	Zymosan	Fungi	[26]	versican	[27]
	Glycosylphosphatidylinositol anchors	Trypanosoma	[28]	Hyaluronan fragments	[29]
	Hemagglutinin protein	Measles virus	[30]		

TLR3	Double-stranded RNA	virus	[31]	mRNA	[32]
	Poly(I:C)		[31]		
	Small interfering RNAs		[33]		

TLR4	LPS	Gram− bacteria	[34, 35]	HSP60, HSP70, HSP72, HSP22, gp96	[15, 18–20, 36–39]
				HMGB1	[22]
				fibronectin, biglycan, tenascin-C, and versican	[25, 40, 41]
				Hyaluronan, lower molecular weight HA, and heparin sulfate	[42–44]

TLR5	flagellin	Bacteria flagella	[45]		

TLR7	Single-stranded RNA		[46]		
	Imidazoquinoline compounds	virus	[47]	ssRNA	[48]
	Guanine analogs		[49]		

TLR8	Single-stranded RNA	virus	[50]	ssRNA	[48]

TLR9	Unmethylated CpG DNA motif	Bacteria, virus	[51]	Chromatin-IgG complex	[52]

TLR11	Profilin-like molecule	Toxoplasma gondii	[53]		
		Uropathogenic bacteria	[54]		

**Table 2:** Role of TLRs in myocardial inflammation and injury after acute ischemia/reperfusion.

	Mice strains	I/R models	I/R protocols	MI	Cardiac function	Inflammation						
TLRs						NF-*κ*B activity	Cytokine production			Myocardial neutrophil infiltration	EC dysfunction/ROS ↑	Ref.
							Myocardium		Serum			
							mRNA	Protein				
TLR2	TLR2^−/−^, TIRAP^−/−^	*ex vivo*	30′ I/60′ R		↑			TNF*α*, IL-1*β*				[86]
	TLR2^−/−^	*in vivo*	30′ I/1 h R	↓			IL-1*β*			↓	↓	[87]
	TLR2^−/−^ chimeric, WT with Anti-TLR2	*in vivo*	30′ I/24 h R	↓	↑			TNF*α*, IL-1*β*, IL-10, M-CSF		↓		[88]
	TLR2^−/−^, WT with Anti-TLR2	*in vivo*	20′ I/24 h R	↓						↓		[89]

TLR4	C57BL/10 ScCr C3H/HeJ	*in vivo*	60′ I/24 h R	↓						↓		[90]
	C3H/HeJ	*in vivo*	60′ I/2 h R	↓		↓	TNF*α*, IL-1*β*, MCP-1, IL-6					[91]
	WT with Eritoran	*in vivo*	30′ I/2 h R	↓			TNF*α*, IL-1*β*, MCP-1, IL-6, MIP-1, MIP-2					[92]
	C3H/HeJ	*in vivo*	60′ I/2 h, 24 h R	↓	NS		TNF*α*, IL-1*β*, IL-6		TNF*α*, IL-1*β*, IL-6			[93]
	C57BL/10 ScCr C3H/HeJ	*ex vivo*	20′ I/60′ R		↑	↓		TNF*α*, IL-1*β*				[94]
	WT with Anti-HSC70	*ex vivo*	20′ I/60′ R		↑		TNF*α*, IL-1*β*, IL-6	TNF*α*, IL-1*β*, IL-6				[95]
	C3H/HeJ	*ex vivo*	HSC70		↑	↓	TNF*α*, IL-1*β*, IL-6	TNF*α*, IL-1*β*, IL-6				[95]
	C3H/HeJ	*ex vivo*	20′ I/60′ R				KC, MCP-1	KC, MCP-1		perfused NE↓		[96]
	C3H/HeJ	*in vivo*	transplantation 2 h I/3 h, 24 h R			↓	TNF*α*, IL-1*β*, IL-6, ICAM-1		TNF*α*, IL-1*β*, IL-6, ICAM-1	↓		[97]

MyD88	WT with Ad-dnMyD88	*in vivo*	45′ I/4 h R	↓		↓						[98]
	MyD88^−/−^	*in vivo*	30′ I/24 h R	↓	↑		MCP-1, KC, ICAM-1			↓		[99]
	MyD88^−/−^	*ex vivo*	20′ I/40′ R	NS	NS							[99]
	MyD88^−/−^ chimeric	*in vivo*	30′ I/24 h R	↓				NS		↓		[100]

Others	CD14^−/−^, MyD88^−/−^, Trif^−/−^	*in vivo*	transplantation 2 h I/3 h, 24 h R			↓	TNF*α*, IL-1*β*, IL-6, ICAM-1		TNF*α*, IL-1*β*, IL-6, ICAM-1	↓		[101]

NS: no significant difference.

**Table 3:** Role of TLRs in myocardial inflammation and injury during viral myocarditis.

TLRs	Mice	Virus	Viral replication	Survival	Myocardial injury marker cTnI	Cardiac function	Inflammation							Ref.
							Myocarditis (pathological change)	Cytokine production					NF-*κ*B activation	
								Myocardial		Serum	IFN-*γ*	IFN-*β*		
								mRNA	Protein					
TLR3	TLR3^−/−^	EMCV	↑	↓	↑		↓	TNF*α*, IL-1*β*, IL-6, RANTES, IP-10↓, IFN-*β*↑				↑		[107]
	TLR3^−/−^	CVB3	↑	↓			↑	IL-12p40, IL-1*β*, IFN-*γ*↓, IFN-*β* NS			↓	NS		[108]
	TLR3^−/−^	CMV	NS		NS		NS							[109]
	TLR3^−/−^	CVB4	↑	↓	↑		↑			TNF*α* and IFN-*α*↓, IL-6 and IFN-*γ* NS	NS			[110]

TLR4	C3H/HeJ (BALB/c)	CVB3	d2↑d12↓				↓		IL-1*β*, IL-18↓, TNF*α*, IFN-*γ*, IL-12p40 NS		NS		↓	[111]

TLR7	TLR7^−/−^	CMV	NS		NS		NS							[109]

TLR9	TLR9^−/−^	CVB3	NS			↑	↓	TNF*α*, TGF-*β*, ICAM-1↓, IFN-*β*↑				↑	↓	[112]
	TLR9^−/−^	CMV	↑		NS		↑							[109]

MyD88	MyD88^−/−^	CVB3	↓	↑			↓	IL-1*β*, TNF*α*, IFN-*γ*, IL-10 and IL-18↓, IFN-*α* and IFN-*β*↑		IL-1*β*, TNF*α*, IFN-*γ*, IL-2, IL-6 and IL-10↓, IL-4 and IL-10 NS	↓	↑		[113]
	MyD88^−/−^	CMV	↑		NS		↑							[109]
	MyD88^−/−^	CVB4	NS	NS	NS		NS			TNF*α* NS, IFN-*α*, IL-6 and IFN-*γ*↓	↓			[110]

Trif	Trif^−/−^	CVB3	↑					IL-12p40, IFN-*γ*↓, IFN-*β* NS			↓	NS		[108]
	Trif^−/−^	CVB3	↑	↓		↓	↑	TNF*α*, IL-1*β*, IL-10, IL-18↑	TNF*α*, IL-1*β*, IL-18↑			d3↑, d7↓		[112]

NS: no significant difference.

## Abbreviations

AAR: Area-at-risk
AP-1: Activator protein 1
BCG: Bacille Calmette-Guérin
CLP: Cecum ligation and puncture
CpG: Cytidine-phosphate-guanosine
CV: Coxsackievirus
DAMPs: Damage-associated molecular patterns
dsRNA: Double-stranded RNA
EAM: Experimental autoimmune myocarditis
ECM: Extracellular matrix
EMCV: Encephalomyocarditis virus
ERK: Extracellular signal regulated kinase
HA: Hyaluronan
HMGB1: High-mobility group box-1
HSP: Heat shock protein
I/R: Ischemia/reperfusion
IFN: Interferon
IKK: I-*κ*B kinase
IL: Interleukin
IRAK: IL-1 receptor-associated kinase
IRF: Interferon regulatory factor 3
JNK: C-Jun N-terminal kinase
KO: Knockout
LPS: Lipopolysacharide
LV: Left ventricle
Mal: MyD88-adaptor like protein
MAPKs: Mitogen-activated protein kinases
MCP-1: Monocyte chemotactic factor-1
MD-2: Myeloid differentiation factor-2
MI: Myocardial infarction
MIP: Macrophage inflammatory protein
MyD88: Myeloid differentiation factor 88
NF-*κ*B: Nuclear factor kappa B
PAMPs: Pathogen-associated molecular patterns
PKC: Protein kinase C
poly(I:C): Polyinosine-polycytidylic acid
PRRs: Pattern recognition receptors
RIP1: Receptor-interacting protein 1
ROS: Reactive oxygen species
SARM: Sterile *α*- and armadillo-motif-containing protein
ssRNA: Single-stranded RNA
TAB: TAK binding protein
TAK1: Transforming growth factor-*α* activated kinase 1
TBK1: TRAF family member-associated NF-*κ*B activator (TANK) binding kinase-1
TIR: Toll/interleukin-1 receptor
TIRAP: TIR domain-containing adaptor protein
TLRs: Toll-like receptors
TNF*α*: Tumor necrosis factor *α*
TRAF: TNF receptor-associated factor
TRAM: Trif-related adaptor molecule
Trif: TIR domain-containing adaptor inducing IFN-*β*—mediated transcription factor
WT: Wild-type.
